# Force Dependent Biotinylation of Myosin IIA by α-Catenin Tagged with a Promiscuous Biotin Ligase

**DOI:** 10.1371/journal.pone.0122886

**Published:** 2015-03-25

**Authors:** Shuji Ueda, Alexandra M. Blee, Katherine G. Macway, Derrick J. Renner, Soichiro Yamada

**Affiliations:** 1 Department of Agrobioscience, Graduate School of Agricultural Science, Kobe University, Kobe, Japan; 2 Department of Biomedical Engineering, University of California Davis, Davis, CA, 95616, United States of America; Northwestern University Feinberg School of Medicine, UNITED STATES

## Abstract

Tissues and organs undergo constant physical perturbations and individual cells must respond to mechanical forces to maintain tissue integrity. However, molecular interactions underlying mechano-transduction are not fully defined at cell-cell junctions. This is in part due to weak and transient interactions that are likely prevalent in force-induced protein complexes. Using *in situ* proximal biotinylation by the promiscuous biotin ligase BirA tagged to α-catenin and a substrate stretch cell chamber, we sought to identify force-dependent molecular interactions surrounding α-catenin, an actin regulator at the sites of cadherin mediated cell-cell adhesion. While E-cadherin, β-catenin, vinculin and actin localize with α-catenin at cell-cell contacts in immuno-fluorescent staining, only β-catenin and plakoglobin were biotinylated, suggesting that this proximal biotinylation is limited to the molecules that are in the immediate vicinity of α-catenin. In mechanically stretched samples, increased biotinylation of non-muscle myosin IIA, but not myosin IIB, suggests close spatial proximity between α-catenin and myosin IIA during substrate stretching. This force-induced biotinylation diminished as myosin II activity was inhibited by blebbistatin. Taken together, this promising technique enables us to identify force sensitive complexes that may be essential for mechano-responses in force bearing cell adhesion.

## Introduction

In multi-cellular organisms, cell-to-cell junctions are force-bearing and highly dynamic, both critical functional requirements for embryogenesis and tissue homeostasis. Proper cell-cell adhesion requires cells to respond to and withstand the mechanical forces that are exerted from neighboring cells. The actin-myosin contractile network exerts force on the sites of cell-cell adhesion, and is an integral component in strengthening adhesive structures. Therefore, how actin-myosin generated forces alter the protein organization at cell-cell contacts is an important detail in the regulation of cell-cell adhesion.

The role of the actin cytoskeleton in cadherin-mediated cell-cell adhesion has been extensively studied. The cadherins, a family of calcium-dependent cell-cell adhesion proteins, play fundamental roles in cell organization during physiological and pathological processes in multi-cellular organisms. The canonical binding partners, α-catenin and β-catenin, are the key regulatory proteins in the cadherin complex. While β-catenin is a well-known component of Wnt pathway, α-catenin recently emerged as a critical player in regulating the actin network at the sites of cadherin mediated cell-cell adhesion.

Recent studies uncovered a unique mechanism by which α-catenin regulates the actin cytoskeleton. The protein sequence of α-catenin contains an actin binding site at the C-terminus, and an overlapping sequence containing a β-catenin binding site and a homo-dimerization site at the N-terminus. Originally, α-catenin was described as a scaffolding protein that links the cadherin complex to the actin cytoskeleton [1]. Interestingly however, α-catenin’s interaction with β-catenin and the actin filament is mutually exclusive [2]. Furthermore, α-catenin’s affinity towards actin filament is much higher as a homo-dimer rather than as a monomer [3], the homo-dimer of α-catenin inhibits Arp2/3-mediated actin nucleation [3,4], and the absence of α-catenin induces membrane activity [5].

These biochemical analyses suggest that α-catenin is unlikely to stably link the cadherin complex and the actin cytoskeleton. While other actin-binding proteins that localize to the sites of cadherin mediated cell-cell adhesion have been proposed as potential linkers [6], detection of the cadherin-actin linker may be obscured due to the transient nature of those interactions that are difficult to replicate *in vitro*. For example, in live cells, the application of mechanical stress via cadherins stiffens the cell junctions [7] and recruits actin [8,9] and vinculin [9,10], suggesting the presence of dynamic force-induced formation of molecular complexes at the sites of cadherin mediated cell-cell adhesion. In fact, the cadherins appear to be under constant stress based on the FRET based strain sensor tagged to the cadherin cytoplasmic domain [11]. Recently, *in vitro* reconstituted cadherin complex has been shown to interact with actin filament in a force-dependent manner [12]. Together, these data demonstrate the temporary association of the cadherin complex and other proteins with the actin cytoskeleton upon force applications.

One mechanism of force-sensitivity at cell junctions is through mechanically-induced conformational changes that expose cryptic sites. For example, in integrin-mediated cell-extracellular matrix adhesion, a number of proteins possess allosteric properties that are regulated by the application of external force (see reviews by [13,14]). In cadherin-mediated cell-cell adhesion, α-catenin unfolds with an elevated level of myosin II activity and subsequently recruits vinculin [15]. In addition, α-catenin unfolding increases the affinity towards vinculin at a single molecule level [16], and vinculin recruitment to the cadherin complex is required for efficient stress hardening of the junctions [7]. Similar to integrin junctions, these recent studies suggest that cadherin mediated cell-cell adhesion is likely regulated by force-sensitive allosteric properties of junctional proteins, but unlike integrin junctions, we know very little about the dynamics or precise composition of these force-sensitive complexes.

Identification and analysis of mechano-responsive proteins rely on methods that often focus on single proteins, such as the use of conformation-sensitive antibodies, FRET, or single molecule force spectroscopy. In contrast, traditional biochemical analysis relies on stable protein-protein interactions *in vitro* to isolate protein complexes, and thus, the inherent nature of the assay prevents detection of force-dependent protein interactions. An alternative approach to traditional immuno-precipitation is to detect proximal proteins by *in situ* biotin labeling [17]. In this technique, a mutant form of biotin ligase, BirA, is linked to a protein of interest, and any proteins in close proximity will be biotinylated at the primary amines. The degree of biotinylation will be determined by the spatial proximity to mutant BirA and the accessibility to the primary amines of proximal proteins. The biotinylated molecules in the protein complexes are subsequently purified using streptavidin beads regardless of whether the complexes remain intact or not. Therefore, proximal biotin labeling may be ideally suited to identify weak or transient protein interactions.

Using a mutant BirA tagged epithelia specific isoform of α-catenin and a cell stretch device, we sought to identify proteins that may associate with α-catenin in a force-dependent manner. Interestingly, the proteins that co-localize at E-cadherin positive cell-cell contacts with immuno-fluorescence are not always biotinylated, suggesting that the spatial resolution of *in situ* biotin labeling exceeds that of standard optical microscopy. Since the purification of proximal proteins does not rely on the stability of protein interactions between binding partners, it is possible to identify mechanically-induced protein interactions. Our analysis demonstrates *in situ* biotinylation as a potential method to screen force-sensitive protein interactions.

## Results

### Characterization of promiscuous BirA-tagged α-catenin fusion expressing cells

**Fig 1:**
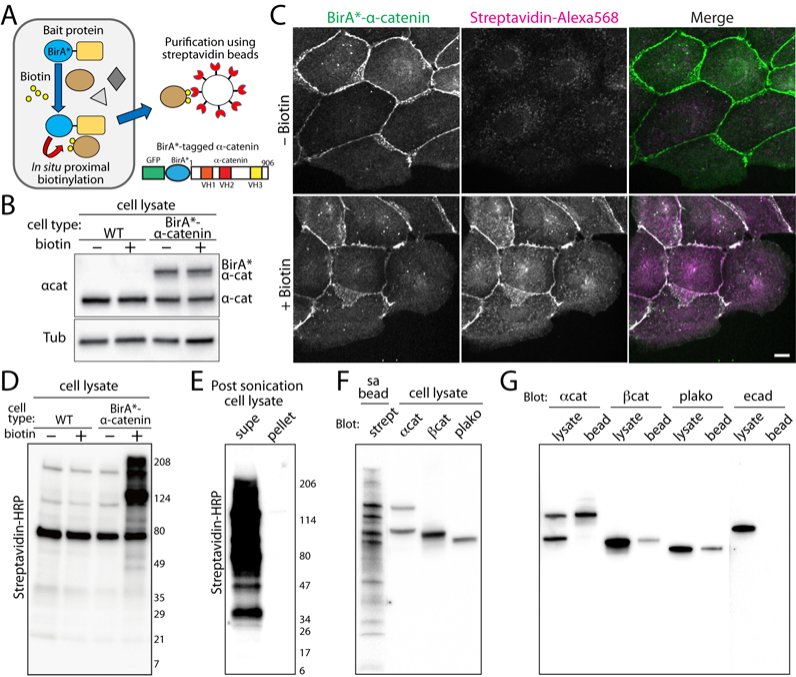
Characterization of promiscuous BirA (BirA*)-tagged α-catenin expressing cells. (A) Illustration of *in situ* proximal biotinylation and subsequent purification of biotinylated proteins. A stable cell line expressing BirA*-α-catenin grown in biotin containing media with subsequent biotinylated proteins purified with magnetic streptavidin-beads. Schematic of a BirA*-α-catenin construct. The BirA* is flanked by GFP and α-catenin. (B) Western blots of the wildtype (WT) and BirA*- α-catenin expressing MDCK stable cell lines. The cell lysates were analyzed using anti-α-catenin (top) and anti-tubulin antibodies (bottom). (C) The BirA*-α-catenin expressing cells were treated with biotin for 24 hours, then analyzed for the localization of BirA*-α-catenin and biotinylated proteins using AlexaFluor-568 labeled streptavidin. BirA*-α-catenin localized to cell-cell contacts, and in the presence of biotin in the media, streptavidin-specific labeling localized to cell-cell contacts, demonstrating the proximal biotinylation by BirA*-α-catenin. Scale bar 20 μm. (D) Detection of biotinylated proteins in BirA*-α-catenin expressing cells. The wildtype (WT) or BirA*-α-catenin expressing stable cell lines were cultured with or without biotin for 24 hours. The cell lysates were analyzed using western blots with streptavidin-HRP. (E) Most biotinylated proteins were in the soluble pool of cell lysates. Post-sonication cell lysates were centrifuged at 16,000g for 20 minutes, and the supernatant and pellet were analyzed using Western blot with streptavidin-HRP. (F) A streptavidin-bead purified sample from cells plated on a P150 dish was analyzed using Western blot with streptavidin-HRP. In adjacent lanes, cell lysate was analyzed using catenin antibodies. The molecular weights of major bands in the purified sample correspond to that of catenins. (G) Cell lysates and purified samples (bead) from cells plated on a P150 dish were analyzed with catenins and E-cadherin antibodies. The catenins were present in the bead fractions but E-cadherin was not.

To identify force-sensitive protein-protein interactions, we designed a fusion protein with a mutant biotin ligase (denoted as BirA*) and epithelia-specific α-catenin. The BirA* contains an arginine to glycine point mutation at residue 118, and has promiscuous biotinylation activity due to the reduced affinity for an intermediate biotin donor, bioAMP [18], which is rapidly released from BirA* and non-specifically reacts with nearby primary amines [17]. The biotinylated proteins are subsequently isolated from cell lysates by streptavidin beads (Fig 1A) [17]. The BirA* sequence was inserted between GFP and α-catenin (Fig 1A), and stably expressed in MDCK cells (Fig 1B).

The BirA*-α-catenin proteins localized to cell-cell contacts in MDCK cells (Fig 1C), and along the lateral domain of cells in confluent cell monolayer (S1A Fig). Despite the over-expression of BirA*-α-catenin, these cells formed morphologically similar cell-cell junctions (Fig 1C and S1A Fig) and assembled into cell aggregates at a similar rate as the wildtype cells (S1B and C Fig). Following incubation with biotin containing media, biotinylated proteins were detected by fluorescently labeled streptavidin (Fig 1C). In the absence of biotin, some vesicular structures were weakly labeled with streptavidin (Fig 1C), likely due to non-specific streptavidin interactions. Interestingly, in the presence of biotin, nuclear staining increased slightly above the background (Fig 1C), suggesting the presence of biotinylated proteins in the nucleus. However, in the presence of biotin, the predominant streptavidin staining was observed at cell-cell contacts (Fig 1C). Furthermore, in the presence of biotin, biotinylated proteins were abundant in cell lysates of BirA*-α-catenin expressing cells, but not in the absence of biotin or in wildtype cells (Fig 1D), thus ensuring that promiscuous BirA biotinylation is the predominant source of biotinylation.

### Purification of biotinylated proteins using streptavidin-conjugated beads

To analyze the proximal biotinylation by BirA*-α-catenin, we first isolated biotinylated proteins from cell lysates using streptavidin conjugated beads. Using cells plated on a standard P150 dish, the cells were scraped and lysed in 0.2% SDS RIPA buffer, then sonicated and centrifuged to separate soluble and insoluble pools. Note that the majority of biotinylated proteins remained in the soluble pool (Fig 1E). Based on previously published protocols, the cell lysates were incubated with streptavidin-conjugated beads in 0.1% SDS containing buffer, then washed with 2% SDS solution to reduce unwanted protein-protein interactions [17]. With concentrated cell lysates from cells cultured in a P150 dish, the purified sample, as detected by streptavidin-HRP, contained several major bands that corresponded to the molecular weights of BirA*-α-catenin, β-catenin, and plakoglobin (Fig 1F). β-catenin and plakoglobin are direct binding partners of α-catenin and also bind to E-cadherin to form the cadherin complex at cell-cell contacts [19]. Using Western blot and with respective antibodies, these catenins were also found in the purified fractions (Fig 1G). Despite a previous study demonstrating that BirA*-tagged E-cadherin biotinylates α-catenin and β-catenin [20], biotinylation of E-cadherin by BirA*-α-catenin was not detectable (Fig 1G). These data demonstrate that the ability of BirA*-α-catenin to biotinylate direct binding partners: β-catenin and plakoglobin.

### Purification of biotinylated protein from cells plated on stretch chambers

**Fig 2:**
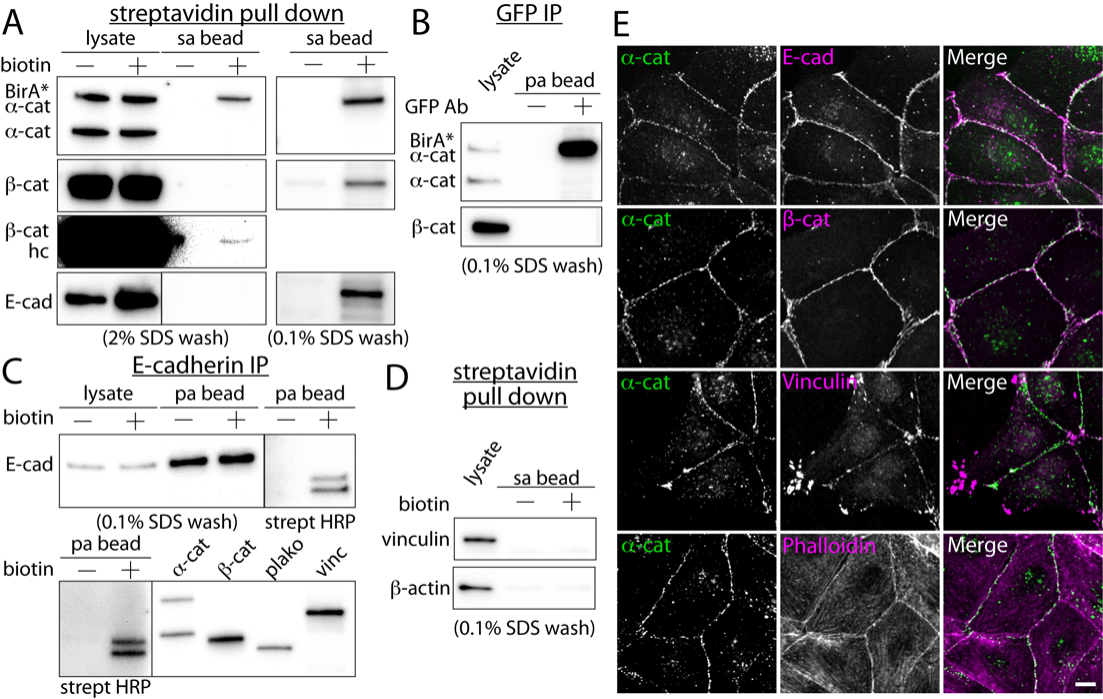
*In situ* proximal biotinylation by promiscuous BirA*-α-catenin. (A) Western blots of purified proteins from BirA*-α-catenin expressing cells using streptavidin-conjugated beads. E-cadherin (E-cad), β-catenin (β-cat), vinculin and β-actin antibodies. Both 2% and 0.1% SDS wash conditions shown. The image contrast of β-catenin blot was enhanced to show the presence of β-catenin in the bead fraction (β-cat hc). (B) Immuno-precipitation of GFP-tagged BirA*-α-catenin proteins using an anti-GFP antibody. The immuno-precipitaed samples were analyzed using Western blot with α-catenin and β-catenin antibodies. (C) Immuno-precipitation of E-cadherin from BirA*-α-catenin expressing cells using an E-cadherin antibody. From immuno-precipitaed proteins, two readily identifiable bands in the streptavidin blot had identical molecular weights as that of β-catenin and plakoglobin. (D) Western blots of strepavidin purified proteins using vinculin and β-actin antibodies. Vinculin and β-actin were not detected in the streptavidin purified protein pool. (E) Co-localization analysis of α-catenin and other cell-cell adhesion and cytoskeletal proteins. While E-cadherin, β-catenin, vinculin and actin filaments co-localize with α-catenin, only E-cadherin and β-catenin are biotinylated by BirA*-α-catenin. Scale bar 10 μm.

We then isolated biotinylated proteins from BirA*-α-catenin expressing cells plated in PDMS stretch chambers in the absence of mechanical perturbations. The challenge of sourcing proteins from cells cultured on the stretch chambers is that the culture surface of the stretch chambers is significantly less than that of standard tissue culture dishes typically used in biochemical analysis (4 cm^2^ vs ~150 cm^2^ of P150 dish). Under the standard purification protocol, BirA*-α-catenin was isolated with the streptavidin-conjugated beads, but endogenous α-catenin was not, consistent with the isolation from a P150 dish (Fig 2A, see also Fig 1F and 1G). In contrast, biotinylation of β-catenin was barely detectable (see the high contrast image of β-catenin blot, Fig 2A, β-cat hc). These data suggest that the degree of biotinylation may affect the efficiency of streptavidin-based purification, especially under a stringent wash condition.

To maximize the biotinylated protein recovery from low protein concentration samples, we tested different SDS concentrations in wash buffer. The high SDS concentration removed significant amount of biotinylated proteins from streptavidin beads (S2A Fig). To retain biotinylated proteins on the streptavidin beads, 0.1% SDS concentration was used to wash the streptavidin-conjugated beads after incubation with cell lysates. With a 0.1% SDS wash solution, α-catenin, β-catenin and E-cadherin came down with the streptavidin-conjugated beads (Fig 2A). Because immuno-precipitation with the GFP antibody isolated GFP-tagged BirA*-α-catenin but not β-catenin under the same condition (Fig 2B), this 0.1% SDS wash is sufficient to disrupt the interaction between α-catenin and β-catenin. This in turn suggests that the presence of β-catenin in streptavidin pull down using 0.1% SDS wash (Fig 2A) is primarily due to the biotinylation of β-catenin, and not α-catenin binding.

Furthermore, using the identical wash condition and cell stretch chambers, immuno-precipitation with the E-cadherin antibody resulted in two bands corresponding to β-catenin and plakoglobin in the streptavidin-HRP blot (Fig 2C), further confirming the biotinylation of β-catenin and plakoglobin, but not E-cadherin (see Fig 1G). Note that this preservation of the interaction between E-cadherin and β-catenin, and not between β-catenin and α-catenin, in 0.1% SDS wash is consistent with the notion that the E-cadherin and β-catenin interaction is much stronger than the β-catenin and α-catenin interaction [21]. The absence of the E-cadherin band in the streptavidin blot of E-cadherin immuno-precipitation samples (Fig 2C) suggests that E-cadherin is not biotinylated. This is consistent with the large-scale purification shown in Fig 1G. Thus, E-cadherin binding to β-catenin is likely responsible for the presence of E-cadherin in streptavidin pull down using 0.1% SDS wash (Fig 2A). These data demonstrate that 0.1% SDS wash minimizes the loss of biotinylated proteins from the streptavidin-conjugated beads, but also preserves the strong protein-protein interactions (e.g. the interaction between E-cadherin and β-catenin), and thus, not all proteins isolated in this procedure will be biotinylated, and biotinylation of purified proteins must be verified with other methods.

In contrast, with 0.1% SDS wash, vinculin and β-actin were not detectable in streptavidin pull down (Fig 2D) despite the accumulation of both vinculin and β-actin at cell-cell contacts (Fig 2E). These data suggest that the promiscuous biotinylation is spatially restricted and co-localization observed by immuno-fluorescence analysis is not a predictor of the BirA* biotinylation.

### Cell stretcher altered cell morphology and actin organization

**Fig 3:**
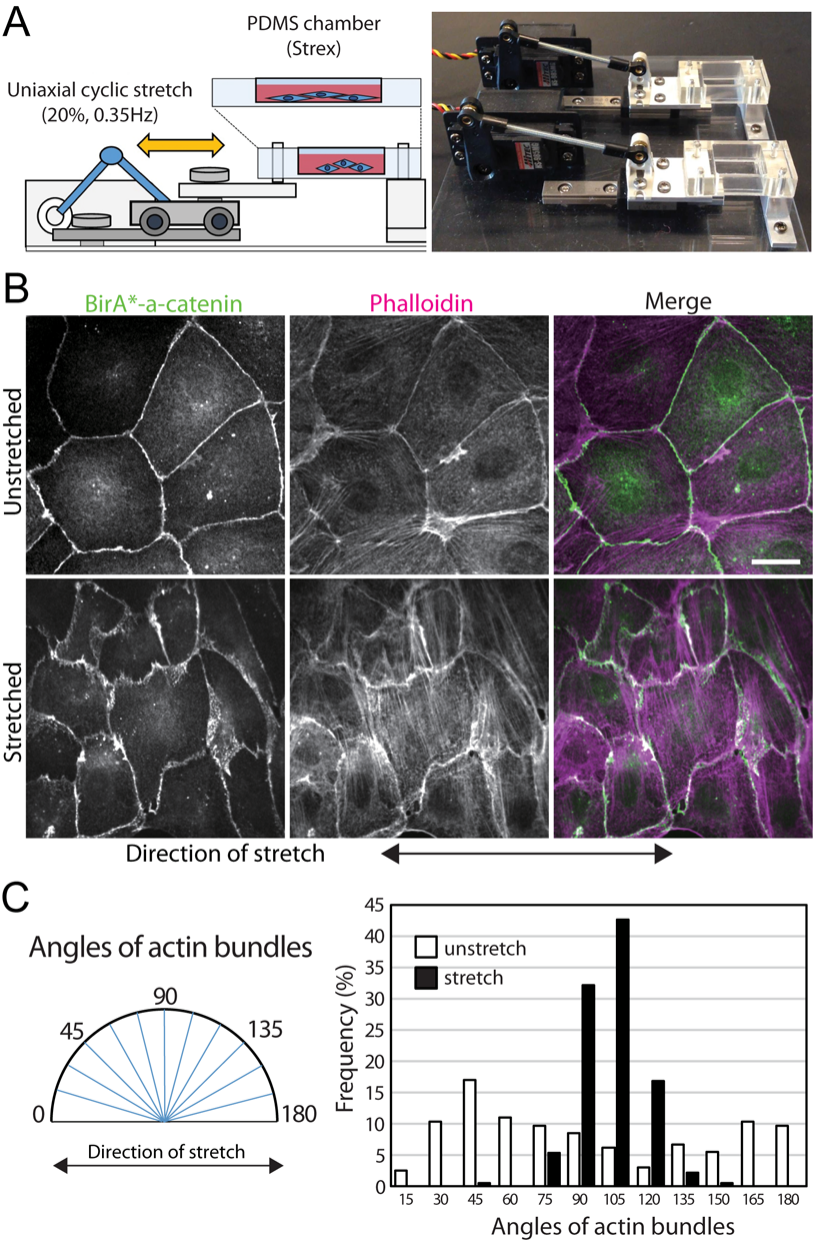
Cyclic stretch induces re-organization of actin fibers. (A) Schematic of the substrate stretch device and an image of the actual device used in this study. The adherent cells in the PDMS cell chamber (STREX) were stretched by a servo motor coupled to the chamber via mechanical linkages. (B) Immunofluorescence analysis of un-stretched or stretched BirA*-α-catenin expressing cells. The actin bundles of the stretched cells are oriented perpendicular to the stretch directions. Scale bar 20 μm. (C) The quantification of the actin bundle rearrangement. The angles are defined as shown in the diagram (left).

Epithelial cell-cell junctions are under constant stress from cell movement. To exaggerate the tension between neighboring cells and induce force-sensitive complexes to assemble, we designed a custom cell stretch device with a PDMS chamber (Fig 3A). Similar approaches have been used to test mechano-responses of various cell types [22–24]. After 12 hours of oscillatory uniaxial stretch (20% at 0.35 Hz), cells elongated perpendicularly to the direction of stretch while α-catenin remained at cell-cell junctions (Fig 3B). In addition, the cells had increased actin stress fibers that aligned perpendicular to the direction of stretch, compared to un-stretched cells (Fig 3B). The quantification of actin bundle orientations demonstrates that most actin bundles oriented perpendicular to the stretch direction, whereas the angles of actin bundles in un-stretched cells were evenly distributed in all directions (Fig 3C). Based on these observations, the custom cell stretch device induces morphological and cytoskeletal changes upon force application.

### Force-induced myosin IIA biotinylation

**Fig 4:**
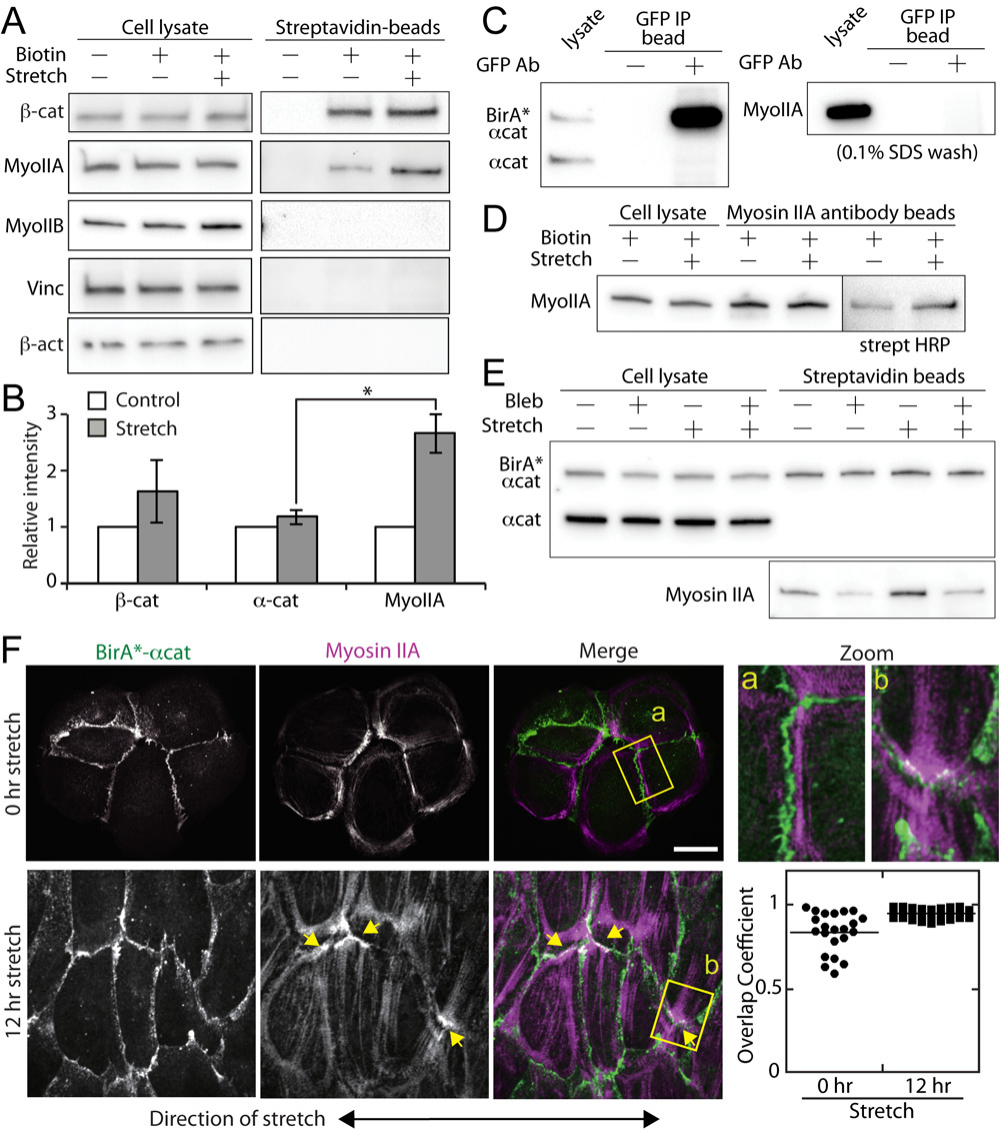
Increased biotinylation of myosin IIA in stretched cells. (A) Western blot analysis of cell lysates and purified streptavidin-conjugated beads from control and stretched cells. With 0.1% SDS wash solution, the purified proteins included β-catenin and myosin IIA. The biotinlyation of myosin IIA increased in mechanically stretched samples. Myosin IIB (MyoIIB), vinculin (Vinc) and β-actin (β-act) were not purified with streptavidin-conjugated beads in unstretched or stretched samples. (B) Quantification of the relative band intensities of unstretched (control) and stretched (stretch) samples. βcat (n = 4), αcat (n = 4) and MyoIIA (n = 7). Results were analyzed using a one-way ANOVA; significance was determined using Dunnett’s post hoc test. Results were considered significant with P<0.05. (C) Immuno-precipitation of GFP-tagged BirA*-α-catenin using a GFP antibody. The immuno-precipitated samples were analyzed with α-catenin and myosin IIA antibodies. The blot of α-catenin is reproduced from Fig 2B. (D) Immuno-precipitation of myosin IIA using a myosin IIA antibody. Immuno-precipitated samples were analyzed with a myosin IIA antibody and streptavidin. (E) Blebbistatin decreases biotinlyation of myosin IIA in both unstretched and stretched samples, suggesting that myosin IIA activity is required for the spatial proximity of α-catenin and myosin IIA. (F) Post-stretching, the cells were fixed and visualized with GFP (BirA*-α-catenin) and anti-myosin IIA antibodies. Yellow arrows point to the end of actin bundles localized at cell-cell contacts where myosin IIA also accumulated. The regions denoted by yellow rectangles (a, b) are magnified in the adjacent images. See also S1 Movie for a 3D stack of stretched cells. Quantification of co-localization between BirA*-α-catenin and myosin IIA was analyzed by calculating overlapping coefficients. Scale bar 20 μm.

In stretched cells, the actin bundles re-oriented and many actin ends inserted into cell-cell contacts (Fig 3B), suggesting the formation of a new protein complex that interacts with the actin cytoskeleton at cell-cell contacts. While the biotinylation level of α-catenin remained unchanged (Fig 4A and 4B), the biotinylation level of β-catenin was variable with a slight increase with the application of substrate stretch (Fig 4A and 4B).

Since the actin-myosin contractile network resists the external forces provided by substrate stretching, we also tested non-muscle myosin IIA, IIB and β-actin for binding to streptavidin-conjugated beads. While the level of myosin IIA was detectable in un-stretched control samples, an increased amount of myosin IIA was observed in stretched samples (Fig 4A and 4B). In contrast, myosin IIB and β-actin were not detectable in both control and stretched samples (Fig 4A). Interestingly, vinculin, a protein thought to be recruited by α-catenin in a force-dependent manner [15], was not detectable in control or stretched samples (Figs 2D and 4A).

Since the biotinylated proteins were purified with 0.1% SDS wash solution, the increased amount of myosin IIA under the stretch condition (Fig 4A) may be explained by an increased affinity toward biotinylated proteins, rather than the elevated level of biotinylation of myosin IIA under mechanical perturbation. Using GFP antibody and 0.1% SDS wash solution, GFP-tagged BirA*-α-catenin was immuno-precipitated (Figs 2B and 4C). While BirA*-α-catenin efficiently came down with the GFP antibody bound beads (Figs 2B and 4C), myosin IIA was not detectable in the bead fraction (Fig 4C). Furthermore, using a myosin IIA antibody, myosin IIA was immuno-precipitated and analyzed for its streptavidin reactivity (Fig 4D). Immuno-precipitated myosin IIA bands reacted with streptavidin, suggesting that myosin IIA was biotinylated (Fig 4D). Similar to the streptavidin pull down results (Fig 4A), streptavidin reactivity increased in the stretch condition relative to the control in the myosin IIA immuno-precipitated samples (Fig 4D), suggesting that increased biotinylation of myosin IIA is a likely explanation for the observed myosin IIA increase in streptavidin pull down with mechanical perturbation (Fig 4A).

In immuno-fluorescence analysis, myosin IIA localized closely, but often adjacent to cell-cell contacts in control samples. However, in the stretched samples, myosin IIA was often recruited to the cell-cell contacts (Fig 4F). This force-dependent recruitment of myosin IIA to α-catenin required myosin IIA activity as blebbistatin, a myosin IIA inhibitor, diminished the biotinylation levels in both control and stretched samples (Fig 4E). Together, these data demonstrate that external forces recruit myosin IIA to α-catenin as observed by increased biotinylation, and that this recruitment depends on myosin II activity.

## Discussion

Actomyosin contractility is an integral part of cadherin-mediated cell-cell adhesion. Previous studies have shown that the absence of myosin IIA disorganizes cadherin mediated cell-cell adhesion [25], and cadherins are at least partially required for recruitment of myosin IIA to cell-cell adhesion [26,27]. Additionally, contractility of myosin II is essential for expansion of cell-cell contacts [28,29]. In our study, substrate stretching induces actin bundles to orient perpendicular to the sites of cell-cell adhesion (Fig 3) and myosin IIA accumulates along cell-cell contacts where these actin bundles terminated (Fig 4C). Since this unique morphology of adherens junctions depends on myosin II activity [30,31], these junctions are thought to be force-bearing junctions, a likely consequence of the substrate stretching.

Our analysis demonstrates surprisingly close interactions between α-catenin and myosin IIA. Under substrate stretching, the BirA*-α-catenin biotinylates myosin IIA more than under a control, un-stretched condition, while the biotinylation level of catenins remain relatively similar regardless of mechanical perturbations (Fig 4D). Interestingly, myosin IIB, a protein related to myosin IIA but with a distinct function at cell-cell adhesions [27], is not biotinylated by BirA*- α-catenin (Fig 4B). In fact, myosin IIA, and but not myosin IIB, plays predominant roles in 3D cell migration [32] and traction force generation in these cells [33]. These results suggest that, under the mechanically enhanced conditions, more myosin IIA proteins are closely associated with α-catenin than in the control, un-stretched condition. In addition, this result raises a possibility that the recruitment of myosin IIA is a direct consequence of force-induced interaction with the cadherin complex rather than an indirect consequence of actin accumulation at force-bearing cell-cell adhesion sites.

While there is no evidence to suggest the direct binding between α-catenin and myosin IIA thus far, such an interaction remains possible as the traditional, force-free solution biochemistry is not designed to test force-induced protein interactions (e.g., Fig 4C). Note, however, that myosin IIA is biotinylated in force-free conditions, albeit less than under the force-bearing conditions (Fig 4). This implies that α-catenin and myosin IIA are in relatively close proximity even in force-free conditions, yet the interaction between them remains undetectable with traditional approaches, suggesting that the α-catenin and myosin IIA interactions are either weak or transient. This is consistent with the observations that we did not detect strong co-localization of α-catenin and myosin IIA in immuno-fluorescence analysis in force-free conditions (Fig 4).

Although it remains possible that myosin IIA biotinylation by BirA* may occur outside the cell-cell contacts, because of increased co-localization between α-catenin and myosin IIA (Fig 4F), we speculate that the increased biotinylation of myosin IIA is in part due to the recruitment of myosin IIA to the sites of cadherin-mediated cell-cell adhesion. This unexpected proximal interaction between α-catenin and myosin IIA raises an intriguing possibility that myosin IIA is a contractile linker between the cadherin complex and the actin cytoskeleton under force-bearing conditions. Previously, the forces generated by myosin IIA are thought to be indirectly distributed to cell-cell junctions. In this case, the actin-myosin contractile network generates forces, which in turn are transmitted to the cadherin complex via the linkers, e.g., EPLIN [34] or α-catenin under force bearing conditions [12]. The results of this study suggest an alternative model of force transmission to the cadherin complex. For example, myosin IIA may directly interact with the cadherin complex while simultaneously binding to the actin cytoskeleton via its motor domain to exert a contractile force along cell junctions.

Interestingly, while α-catenin is a direct binding partner of vinculin, which localizes to the cell-cell contacts of MDCK cells (Fig 2E), vinculin was not biotinylated in our assays (Figs 2D and4A). This is not due to a poor vinculin antibody reaction as the vinculin antibody has a similar sensitivity to the α-catenin antibody (S2B Fig). Vinculin is recruited to the sites of cadherin mediated cell-cell adhesion by the application of force via cadherins [7,9,10] and vinculin localization to cell-cell contacts is reduced by myosin II inhibitors [15,35]. This unique force dependent recruitment is thought to be mediated by a force-induced conformational change of α-catenin that exposes the cryptic vinculin binding site of α-catenin [15]. In MDCK cells, however, contractility-dependent vinculin recruitment to cell-cell contacts is not as robust as other cell types [15,36], although vinculin is recruited to force-bearing cadherin complexes in the purse string actin network during single cell wound healing of a MDCK cell monolayer [15,36]. However, deletion of the vinculin binding domain of α-catenin clearly diminishes vinculin accumulation at cell-cell contacts of MDCK cells [37].

It may be possible that either BirA* tagged to the N-terminus of α-catenin is unable to biotinylate α-catenin bound vinculin or the force-induced conformational change in α-catenin may somehow prevent vinculin biotinylation. This is consistent with the observation that β-catenin and plakoglobin are robustly biotinylated by BirA* because they interact with the N-terminus of α-catenin, but actin, which interacts with the C-terminus of α-catenin, is not biotinylated. Therefore, the relative location of BirA* to α-catenin likely dictates the biotinylation profile, and further studies are needed to unravel the complete list of proximal proteins.

Our approach to use proximal biotinylation as a means to detect force-induced formation of protein complexes is based on screening of known proteins that localize to the sites of cadherin-mediated cell-cell adhesion. Thus, this approach is limited to prior knowledge of proteins previously identified at sites of cell-cell adhesion, though many adherens junction-associated proteins are already identified. Alternatively, previous studies have combined proximal biotinylation and subsequent streptavidin-based purification with mass spectrometry in the analysis of the nuclear pore complex [17,38] and adhesive structures [20,39]. Furthermore, this proteomic approach has identified contractility dependent recruitment of LIM proteins at focal adhesions [40]. Use of mass spectrometry on biotinylated samples is an attractive methodology, but currently, the size of the flexible chambers limits the number of cells, which is insufficient for detection by mass spectrometry. Nevertheless, our current study validates proximal biotinylation as a methodology to identify force-sensitive protein interactions, and future studies will focus on the scaling-up of substrate stretching devices to establish a list of biotinylated force-sensitive proteins.

## Materials and Methods

### Reagents

For western blot or immuno-fluorescence applications, the following antibodies were used: GFP (rabbit polyclonal, Invitrogen, Carlsbad, CA USA), αE-catenin (15D9, Alexis Biochemical, Farmingdale, NY, USA), α-tubulin (DM1A, Sigma-Aldrich, St. Louis, MO, USA), E-cadherin (clone 36, BD Bioscience, San Jose, CA, USA), β-catenin (clone 14, BD Biosciences), non-muscle myosin IIA (rabbit polyclonal, Sigma), vinculin (hVIN-1, Sigma) and β-actin (AC-15, Sigma). Filamentous actin was labeled with AlexaFluor-568-conjugated phalloidin (Invitrogen), and biotinylated proteins were labeled with AlexaFluor-568-conjugated streptavidin. For western blotting, the signals on the nitrocellulose membrane were detected using a Western Quantum Bright chemiluminescence kit (Advansta, CA, USA). Following pharmacological reagents were used in this study: biotin (Sigma), (-)-blebbistatin (Calbiochem, San Diego, CA, USA), and cComplete protease inhibitor cocktail (Roche, Indianapolis, IN, USA).

### Plasmid constructs and the stable cell lines

MDCK cells were cultured in Dulbecco’s modified Eagle’s medium (Invitrogen) supplemented with 10% fetal bovine serum (Atlanta Biologicals, GA, USA), penicillin and streptomycin (Invitrogen). The mutant BirA R118G (BirA*, Addgene, Cambridge, MA, USA) was PCR amplified using the following primers: 5’-CGAGCTCAAGCTTCGAAGGACAACACCGTGCCC (Forward) and 5’-GCAGTCATGGTGGCGGCCTTCTCTGCGCTTCTCAGG (Reverse), then, using SalI and EcoRI, the PCR product was inserted between GFP and epithelial-α-catenin in the pEGFP-C1-α-catenin vector. The GFP-BirA*-α-catenin sequence was then inserted into the PiggyBac vector using NheI and SacII sites (PB533A, System Biosciences, Mountain View, CA). The Piggybac GFP-BirA*-α-catenin plasmid and Piggybac Transposase expression plasmid (System Biosciences) were transfected into MDCK cells using Lipofectamine2000 (Invitrogen) and selected with 500 μg/ml G418 (Invitrogen). G418-resistant cells were sub-cloned and selected by confocal microscopy and western blotting to obtain homogeneous cell populations.

### Immuno-fluorescence and confocal microscopy analysis

MDCK cells were fixed using either 1.5% (for myosin IIA staining) or 3% (for all others) para-formaldehyde and 0.3% Triton X-100 in PBS for 10 minutes and blocked with 1% BSA and 0.3% Triton X-100 in PBS. Primary antibodies were added and incubated for 1 hour at room temperature, and detected using AlexaFluor-568-conjugated secondary antibodies or AlexaFluor-568-conjugated streptavidin (Invitrogen). Samples were imaged with a Zeiss Axio Observer equiped with Yokogawa spinning confocal system (CSU-10), 40x C-Apochromat water immersion objective, 488/561 nm solid state laser system, and a CoolSNAP HQ camera. The microscope system was controlled by Slidebook software (Intelligent Imaging Innovations, Denver, CO, USA). The alignment of actin bundles was manually analyzed using ImageJ. The overlap coefficients for co-localization analysis of α-catenin and myosin IIA were calculated based on Manders et al. [41]. If the overlapping coefficient of two proteins is 1, then they are perfectly co-localized, and if the overlapping coefficient of two proteins is 0 then they are not.

### Cell stretching

Mechanical stretch device consists of a linear stage for uniaxial stretch driven by a servo motor (HiTec, CA, USA) and a servo controller (Pololu, NV, USA) with a customized stage for stretch chambers (Fig 3A). BirA*-α-catenin expressing cells were seeded on 20×20mm PDMS (polydimethylsiloxane) membrane chamber (STREX, Osaka, Japan) coated with collagen type I. After 24 hours, cells were subjected to uniaxial cyclic stretch (20%, 0.35Hz) in a standard tissue culture incubator maintained at 37°C with 5% CO_2_.

### Purification of biotinylated proteins and immuno-precipitation

Cells were seeded 2×10^5^ cells on p150 dishes or 2×10^5^ cells on PDMS membrane chamber and cultured for 20–24 hours in complete media supplemented with 50 μM biotin, and if indicated, 50 μM blebbistatin. After PBS washes, cells were lysed in RIPA buffer (25 mM Tris–HCl pH 7.5, 150 mM NaCl, 1% Triton-X 100, 0.2% SDS, 0.5% sodium deoxycholic acid, 1 mM DTT, protease inhibitor) and sonicated (duty ratio 50%, duration 30 seconds to 1 minute) on ice. After centrifugation at 16.1x10^3^g for 20 min, cell supernatants were collected as a lysate sample. Protein concentrations were measured by Bradford assay. The lysates were diluted with TN buffer (150 mM NaCl and 25 mM Tris–HCl pH 7.5) to decrease the SDS concentration to 0.1%. The diluted lysates were incubated with magnetic streptavidin-beads (MyOne Streptavidin C1, Invitrogen) for 1.5 hours. Using magnetic collection stands, streptavidin-beads were washed with RIPA buffer with either 2% or 0.1% SDS. In some experiments, the samples were further washed with wash buffer 2 (0.1% deoxycholate, 1% Triton-X 100, 500 mM NaCl, 1 mM EDTA, and 50 mM Hepes, pH 7.5), wash buffer 3 (250 mM LiCl, 0.5% NP-40, 0.5% deoxycholate, 1 mM EDTA, and 10 mM Tris, pH 8.1) and wash buffer 4 (50 mM Tris, pH 7.4, and 50 mM NaCl) for one time each, but similar results were obtained regardless. Purified proteins were dissolved in SDS sample buffer and separated by SDS-PAGE in a 4–15% gradient precast gel (Bio-Rad, Hercules, CA, USA). The identical protocol was used for immuno-precipitation using E-cadherin, GFP, and myosin IIA antibodies except that antibodies were cross-linked to Protein A magnetic beads (Invitrogen).

## Supporting Information

S1 Fig Characterization of BirA*-α-catenin expressing MDCK cells. (A) Subcellular localization of BirA*-α-catenin and E-cadherin in sub-confluent and confluent cell monolayer. The overlay images of BirA*-α-catenin and E-cadherin staining are shown in the last panel (Merge). For the confluent cell monolayer, 3D stack images were reconstructed to visualize the lateral membrane localization of BirA*-α-catenin and E-cadherin. Scale bar 10 μm. (B) Hanging drop assay to test cell aggregation potential of wildtype and BirA*-α-catenin expressing cells. Cells were suspended at a density of 2.5 x 10^5^ cells/ml medium. 25 µl of cell suspension was seeded onto glass-bottom dishes, inverted upside-down, and incubated at 37°C. Cell suspensions were then triturated through a pipette tip 30 times and the cluster sizes were quantified using ImageJ. The data are represented as mean cluster size ± standard error of the mean.
(TIF)

S2 Fig Dependence of SDS concentration and antibody sensitivity. (A) The SDS concentration in wash buffer. Streptavidin-conjugated beads were washed with solutions containing different SDS concentration (%), then the wash solutions and the bead fractions for each SDS concentration were collected and analyzed using Western blot with streptavidin–HRP. While high SDS concentrations (0.5–2%) removed a significant amount of biotinylated proteins from the beads, the removal of biotinylated proteins were minimal for 0.1% SDS wash solution. (B) Relative detection sensitivity of α-catenin and vinculin antibodies. The lysates from MDCK cells expressing GFP-tagged α-catenin or vinculin were loaded onto a SDS-gel and analyzed with Western blot using GFP, α-catenin or vinculin antibodies. The blot analyzed with the GFP antibody shows relative loading of GFP-tagged proteins (left). The same sample volumes were loaded onto the adjacent lanes and analyzed with α-catenin or vinculin antibodies under the identical exposure of the blot (right). The identical antibody dilution as main figures (1:1000 for both antibodies) was used in this experiment. The α-catenin and vinculin antibodies detected the exogenous GFP-tagged α-catenin and vinculin, respectively, as well as the endogenous proteins. The relative intensities of GFP-tagged proteins in the GFP blot (left) and α-catenin or vinculin blot (right) are similar, suggesting that the detection sensitivity of vinculin antibody is similar to that of α-catenin antibody. Therefore, the lack of vinculin bands in streptavidin bead purified samples (see Fig 2A and 4B) is not simply due to poor sensitivity of the vinculin antibody.
(TIF)

S1 Movie A 3D stack of stretched samples shown in Fig 4F. The images were taken at 0.5 micron spacing denoted by the values in the upper left corner.
(MOV)
