# Supplementary figures and images for: Force Dependent Biotinylation of Myosin IIA by α-Catenin Tagged with a Promiscuous Biotin Ligase
Source: PLoS One. 2015 Mar 25;10(3):e0122886. doi: 10.1371/journal.pone.0122886 (PMC4373798; doi:10.1371/journal.pone.0122886)

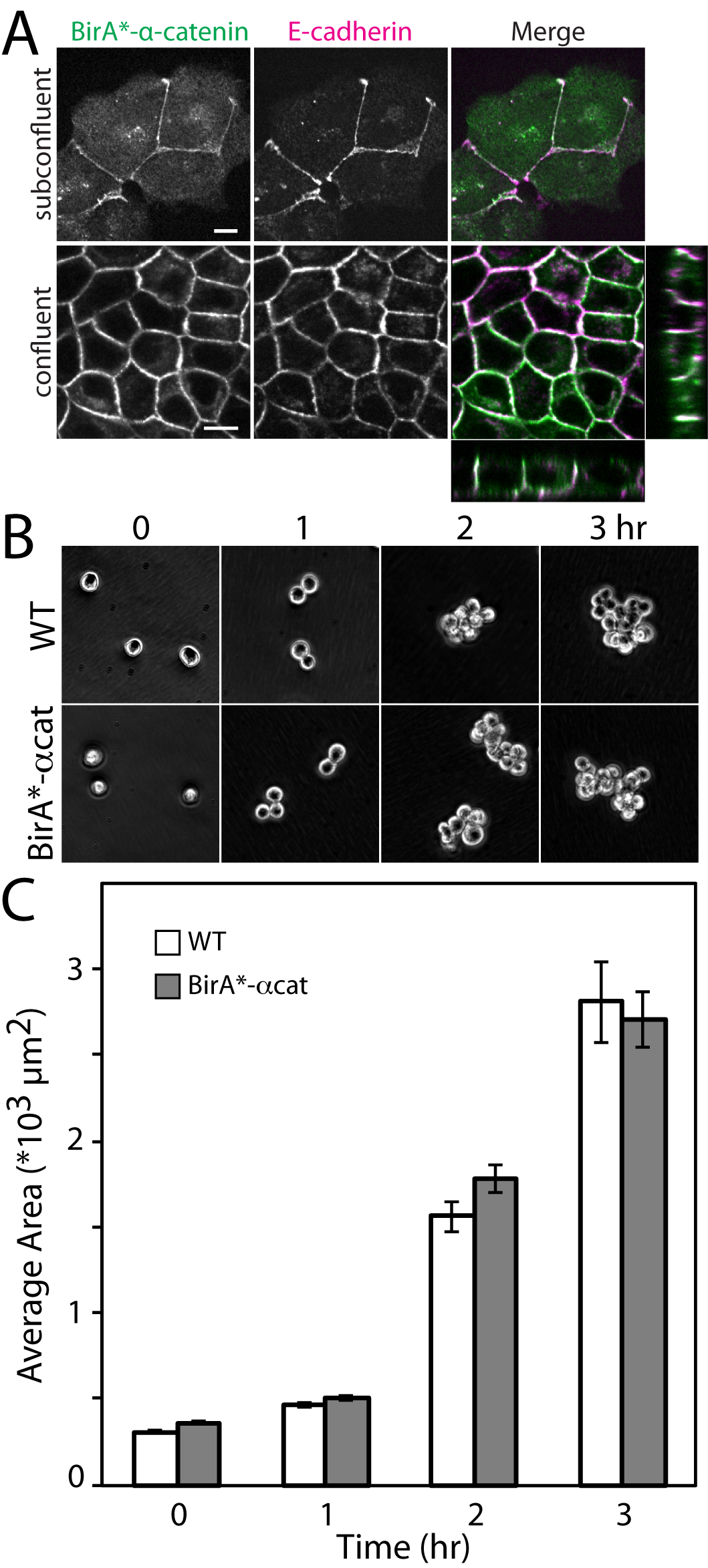

Supplement: S1 Fig — (A) Subcellular localization of BirA*-α-catenin and E-cadherin in sub-confluent and confluent cell monolayer. The overlay images of BirA*-α-catenin and E-cadherin staining are shown in the last panel (Merge). For the confluent cell monolayer, 3D stack images were reconstructed to visualize the lateral membrane localization of BirA*-α-catenin and E-cadherin. Scale bar 10 μm. (B) Hanging drop assay to test cell aggregation potential of wildtype and BirA*-α-catenin expressing cells. Cells were suspended at a density of 2.5 x 10^5^ cells/ml medium. 25 µl of cell suspension was seeded onto glass-bottom dishes, inverted upside-down, and incubated at 37°C. Cell suspensions were then triturated through a pipette tip 30 times and the cluster sizes were quantified using ImageJ. The data are represented as mean cluster size ± standard error of the mean. (TIF) [file pone.0122886.s001.tif]

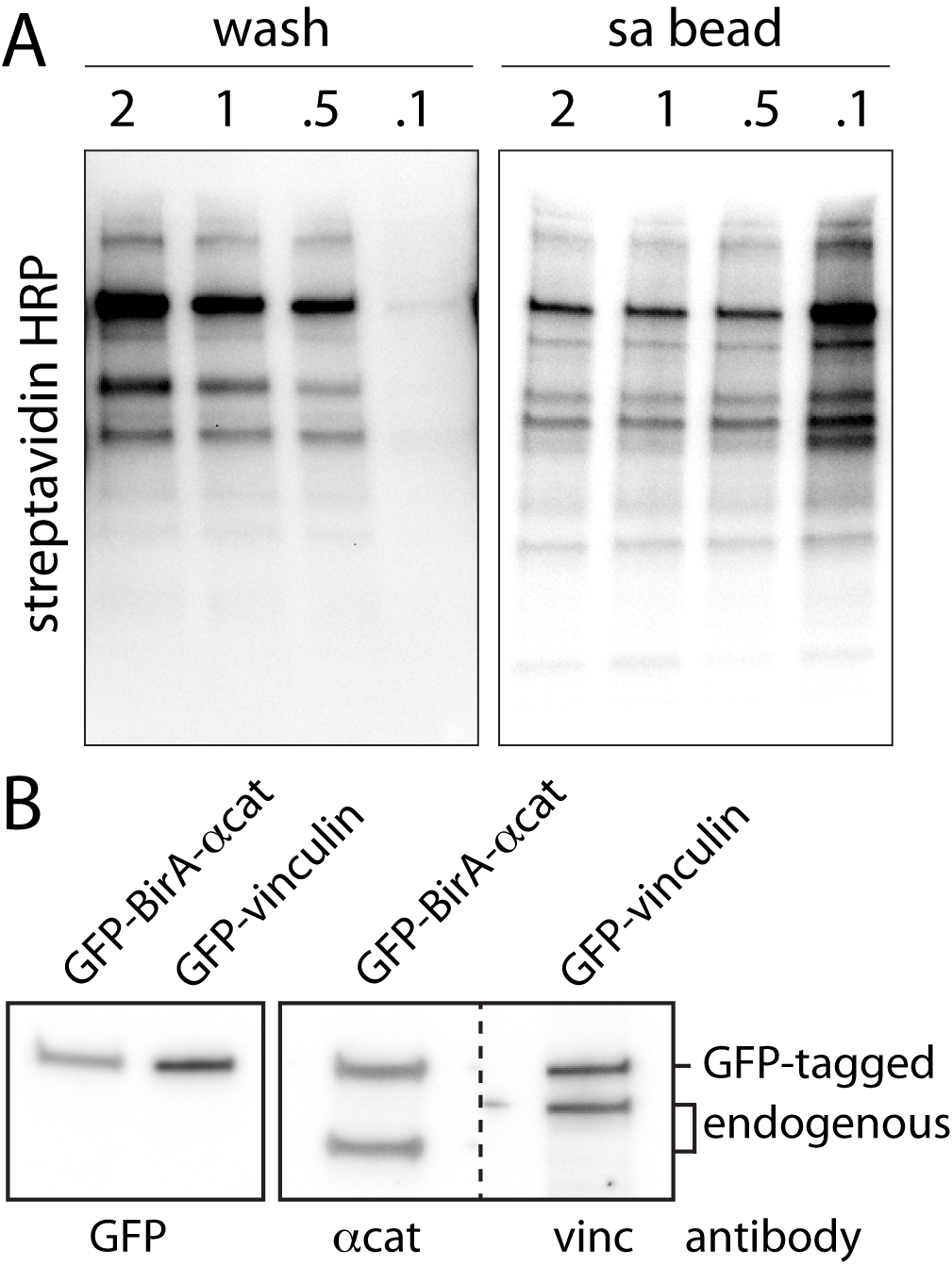

Supplement: S2 Fig — (A) The SDS concentration in wash buffer. Streptavidin-conjugated beads were washed with solutions containing different SDS concentration (%), then the wash solutions and the bead fractions for each SDS concentration were collected and analyzed using Western blot with streptavidin–HRP. While high SDS concentrations (0.5–2%) removed a significant amount of biotinylated proteins from the beads, the removal of biotinylated proteins were minimal for 0.1% SDS wash solution. (B) Relative detection sensitivity of α-catenin and vinculin antibodies. The lysates from MDCK cells expressing GFP-tagged α-catenin or vinculin were loaded onto a SDS-gel and analyzed with Western blot using GFP, α-catenin or vinculin antibodies. The blot analyzed with the GFP antibody shows relative loading of GFP-tagged proteins (left). The same sample volumes were loaded onto the adjacent lanes and analyzed with α-catenin or vinculin antibodies under the identical exposure of the blot (right). The identical antibody dilution as main figures (1:1000 for both antibodies) was used in this experiment. The α-catenin and vinculin antibodies detected the exogenous GFP-tagged α-catenin and vinculin, respectively, as well as the endogenous proteins. The relative intensities of GFP-tagged proteins in the GFP blot (left) and α-catenin or vinculin blot (right) are similar, suggesting that the detection sensitivity of vinculin antibody is similar to that of α-catenin antibody. Therefore, the lack of vinculin bands in streptavidin bead purified samples (see Fig 2A and 4B) is not simply due to poor sensitivity of the vinculin antibody. (TIF) [file pone.0122886.s002.tif]
